# Correlates of self-reported and biomarker based adherence to daily oral HIV pre-exposure prophylaxis among a cohort of predominantly men who have sex with men in Nigeria

**DOI:** 10.1371/journal.pone.0282999

**Published:** 2023-03-16

**Authors:** Olusegun A. Adeyemi, Rebecca G. Nowak, Mark Marzinke, Daniel Morgan, Nadia Sam-Agudu, Jaih Craddock, Min Zhan, Trevor A. Crowell, Stefan Baral, Nicaise Ndembi, Sylvia Adebajo, Manhattan E. Charurat, for the TRUST/RV368 Study Group

**Affiliations:** 1 Department of Public Health and Epidemiology, University of Maryland School of Medicine, Baltimore, Maryland, United States of America; 2 International Research Center of Excellence, Institute of Human Virology, Abuja, Nigeria; 3 Institute of Human Virology, University of Maryland School of Medicine, Baltimore, Maryland, United States of America; 4 Department of Pathology, The Johns Hopkins University School of Medicine, Baltimore, MD, United States of America; 5 School of Social Work, University of Maryland, Baltimore, MD, United States of America; 6 Division of Biostatistics and Bioinformatics, Department of Public Health and Epidemiology, University of Maryland School of Medicine, Baltimore, Maryland, United States of America; 7 Henry M Jackson Foundation for the Advancement of Military Medicine, Bethesda, MD, United States of America; 8 U.S Military HIV Research Program, Silver Spring, Maryland, United States of America; 9 The Johns Hopkins Bloomberg School of Public Health, Baltimore, MD, United States of America; 10 Center for International Health, Education, and Biosecurity, Abuja, Nigeria; UNSW Australia, AUSTRALIA

## Abstract

**Introduction:**

HIV pre-exposure prophylaxis (PrEP) significantly reduces the risk of HIV acquisition. However, studies have demonstrated discordance between self-reported measures and biomedical benchmarks of PrEP adherence. We estimated the correlation between self-reported PrEP adherence and PrEP biomarkers and explored factors associated with adherence among men who have sex with men (MSM) in Nigeria.

**Methods:**

TRUST-PrEP, an open-label, prospective study; conducted in Abuja between April 2018 and May 2019. MSM ≥ 18 years with substantial HIV risk were enrolled. Participants reported PrEP adherence in the last month using a 4-point scale from “poor” to “perfect” and serum samples for PrEP biomarkers were collected at months 3 and 9. Serum tenofovir concentration was measured by liquid chromatography-tandem mass spectrometry and considered protective for adherence if ≥ 4.2 ng/ml. Spearman’s rank correlation was used to estimate correlation between self-reported adherence and measured tenofovir levels. Generalized estimating equations with a logit link was used to estimate adjusted odds ratios (aORs) and 95% confidence intervals (CIs) for associations between self-reported adherence and laboratory-measured adherence.

**Results:**

A total of 219 MSM with median age 23 (interquartile range 20–27) years had at least one PrEP biomarker assay. Only 66/219 (30%) had at least one record of protective tenofovir concentration. Correlation between tenofovir and self-reported adherence at 3 and 9 months were 0.1 and 0.02 respectively. Furthermore, 17/219 (8%,) and 49/219 (22%) had serum tenofovir of 4.2–35.4 ng/mL and ≥ 35.5 ng/mL, corresponding to at least 4 and 7 days’ PrEP use in a week, respectively. PrEP adherence was higher among participants introduced to PrEP in the clinics compared with communities (aOR: 8.35, 95%CI: [3.24, 21.5]) and those with same-sex practices family disclosure (aOR: 3.60 95% CI: [1.73, 7.51]).

**Conclusion:**

Self-reported PrEP adherence poorly correlated with biomarkers. Facilitating clinic-based PrEP introduction and disclosure of same-sex practices to family among MSM may improve PrEP adherence.

## Introduction

HIV prevention strategies for men who have sex with men (MSM) are critical for controlling the HIV epidemic [1–3]. In some countries, for example, Nigeria, Kenya and United States MSM bear between 15 to 70% of the burden of all incident HIV infections despite constituting between 3 and 5% [4–6] of the population. Nigeria has the second-largest HIV epidemic globally [7], comprising 8% of the global new HIV infections in 2019 [8]. While intensified and consolidated efforts have resulted in decreased HIV prevalence among the general population over the past two decades [9], HIV prevalence continues to increase among MSM [1, 10]. In comparison to WHO’s recommendation for pre-exposure prophylaxis (PrEP) initiation at HIV incidence of 3 per 100- person-years, HIV incidence among MSM in Nigeria was 5.8–23.1 per 100- person-years in 2020 [11].

MSM living in Nigeria have specific challenges accessing health care services, predominantly attributed to systemic [12–14] and internalized homophobia [15]. In a previous Nigerian study [15], up to a third of MSM reported internalized homophobia, which has been associated with worse health-seeking behaviors [16]. Therefore, increasing PrEP accessibility to MSM in Nigeria, in addition to appropriate risk-reduction messaging and adherence counseling [17], present an opportunity for an additional HIV prevention strategy.

Biomedical chemoprophylaxis via daily oral HIV PrEP has been demonstrated to reduce HIV infection [18, 19]. The most common reason for PrEP implementation failure is poor adherence [18, 19]. Because of the strong association demonstrated between adherence and the success of PrEP implementation programs [18, 20–24] several studies [25–28] have investigated various methods of PrEP adherence measurements over the past few years.

Since the approval of tenofovir disoproxil fumarate/emtricitabine (TDF/FTC) for PrEP by the United States’ Food and Drug Administration in 2012 [29, 30] plasma, dried blood spots [31–33], self-report [25, 26, 28, 33–35], and pill counts have been evaluated and utilized to measure PrEP adherence. A few studies from Sub-Saharan African countries utilized the dried blood spots methods for PrEP adherence assessment among high risk populations for HIV [22, 36]. Furthermore, the, self-reported adherence method was utilized in East Africa among a population with elevated HIV risk [37]. Some previous studies [25, 38, 39] have shown that self-reported PrEP adherence may over-estimate the subjective equivalence; for example, participants of the ATN-110 / ATN-113 studies over reported adherence by 40% (95% Confidence Interval [CI]: 31%, 49%). In contrast, other studies [31, 40, 41] have shown that self-reported PrEP adherence could be a good proxy for PrEP adherence. TDF/FTC “PrEP” adherence benchmarks established by directly observed therapy trials have been widely used [31, 42–44].

Most studies [25, 32, 33] that have investigated the fidelity of self-reported PrEP adherence among MSM have been conducted in high income, and in relatively low HIV burden settings; few have been surveyed in West Africa. Therefore, the previous studies’ generalizability to low-middle income and high HIV burden settings like Nigeria may be limited.

The main objective of this study was to estimate the relationship between self-reported PrEP adherence and systemic concentrations of tenofovir (TFV) and FTC (Main aim). We further explored the systemic concentration of TFV as a binary measure of PrEP adherence (Sub-aim). We hypothesized that there would be a chronologically progressive increase in discordance between self-reported PrEP adherence and PrEP biomarker estimates later compared with earlier study periods, post-PrEP initiation. Within this study, we discussed only Tenofovir Disproxil Fumarate (TDF) and Emtricitabine (FTC) prescribed for daily oral PrEP.

## Materials and methods

### Study design and sample population

In the TRUST-PrEP study, we introduced study participants to PrEP via two methods—peer-to-peer introduction (the community-based method) and provider-initiated introduction (the clinic-based method). For the community-based method, trained popular opinion leaders (POLs) engaged MSM within their communities and networks, discussed PrEP with them, and recruited those interested via the use of coupons with unique identifiers. Each POL was provided three coupons for each wave of recruitment of three MSM from their network. More coupons were rendered to POLs for a maximum of three waves of recruitment; thus, a POL could have a minimum of zero recruitment and a maximum of nine. On presenting to the study clinic, the coupons presented were verified by their serial numbers before the community-recruited MSM were screened and assessed for eligibility for the parent study (TRUST); if eligible, they were required to complete the PrEP study’s willingness questionnaires before assessment for TRUST-PrEP’s eligibility. For the clinic-based method, providers with expertise in sexual and gender minorities discussed PrEP with MSM attending TRUST clinics, and those interested were noted on the patient encounter forms, and further required to complete the willingness questionnaires, screened and assessed for TRUST-PrEP eligibility. All participants received their PrEP prescription from the TRUST clinic and picked their medications from the pharmacy attached to the clinic. TRUST-PrEP enrollment occurred between April 2018 and May 2019, and study follow-up continued until June 2020.

### Study population and eligibility criteria

The study population was MSM residing in Abuja, North-Central Nigeria, and its environs. To be eligible for enrollment in TRUST-PrEP, the participant had to meet the parent study’s (TRUST) criteria. These included "male" assignment at birth, history of receptive or insertive anal sex with another male in the last 12 months, and the ability to provide informed consent in the dominant local language (Hausa) or English. At enrollment, participants presented a valid study coupon, were at least 16 years of age (considered able to access sexual and reproductive health (including HIV) care and research participation without parental consent) [45], were living without HIV, and consented to daily oral PrEP use and biological specimen collection at each visit for the study period. They also had to have met at least one of the following screening criteria for "substantial risk for HIV infection". These included sexual activity with a report of condomless vaginal or anal intercourse with more than one partner, sex partner with HIV risk or recent history of STIs all within the past three months. Other criteria included history of sharing injection or injection materials and history of sexual partner living with HIV within the past three months.

### Data collection

This one-year study consisted of five clinic visits at baseline, months 1, 3, 6, and 9. At baseline, demographic and behavioral information were collected, as well as biological samples (blood, urethral swabs, and rectal swabs) for sexually transmitted infections (HIV, urethral and rectal *Neisseria gonorrhoeae* and *Chlamydia trachomatis*). Biological samples were collected at every visit except month 1. At month 1, an interviewer administered a self-reported 30-day PrEP questionnaire to participants, and this was collected at subsequent visits. PrEP bio-marker assays were collected and post-baseline follow up behavioral questions were asked at months 3 and 9. Stored serum samples were used to determine the concentrations of TFV and FTC. Drug concentrations were determined via liquid chromatographic tandem mass spectrometry (LC-MS/MS) analysis using previously described, validated assays [32, 46]; the assay lower limit of quantification was 0.31 ng/ml for both compounds [32]. We used serum samples because resources for other methods like dried blood spots were not available.

### Ethical approval statement

Ethical clearance was obtained from the Federal Capital Territory Research Ethics Committee, Abuja (FHREC/2012/01/22/09-08-12), and the Institutional Review Board of the University of Maryland, Baltimore (HP-0052013). All participants provided written informed consent prior to any study procedures.

### Inclusivity in global research

Additional information regarding the ethical, cultural and scientific considerations specific to inclusivity in global research is included in the S1 Checklist.

### Study variables

#### Outcome variables

Serum TFV and FTC were measured as both continuous and dichotomized variables. For the main aim, we used a cube-rooted value of the absolute measure, *"cube-rooted ng/ml"*, while for the sub-aim, TFV and FTC adherence were dichotomized at 4.2 ng/mL and 4.6 ng/mL, respectively, based on the 90% sensitivity “inclusive” benchmark of serum concentration for four tablets per week recommended for protective TFV concentration [32]. Serum TFV and FTC concentration equal or higher than 35.5 ng/ml and 49.1 ng/ml are associated with daily use [32]. In this study, we defined protective TFV concentration as serum TFV concentration equal to or higher than 4.2 ng/mL, irrespective of assay time. Furthermore, we defined daily use of PrEP as serum TFV concentration equal or higher than 35.5 ng/ml.

#### Exposure variable

The primary exposure was the 30-day, daily oral PrEP adherence self-report. This was reported on a 4 point Likert scale as *"Poor"*, *"Good"*, *Very good" or "Perfect"* in response to the question *"How would you describe your adherence over the last one month*?*"* asked at every study visit (months 1, 3, 6 and 9). For the main aim, this was analyzed as an ordered category (poor, good, and very good). Very few participants reported, *"perfect*", hence, that response was categorized with "very good". For the sub-aim, this was categorized as a binary variable; self-reported PreP adherence (SRPA =“Poor”) for those that reported “poor” and (SRPA = "Better than poor") for those that reported “perfect, *very good"* or "*good*".

#### Covariates

Demographic variables including age, educational attainment (≤ high school [completed formal secondary school or less] or > high school [completed more than a formal secondary school education]), marital status (never married or ever married), employment status (employed or unemployed) and gender identity (cisgender man or transgender woman). Age was dichotomized into a binary variable: younger (16–24) or older (≥ 25 years). Behavioral variables included sexual orientation (homosexual or bisexual), condom use during last anal sex with a male partner (yes or no), concurrent partnership with two or more male partners (yes or no) and number of alcoholic drinks in the past 30 days ("Alcohol in last 30 days” 0–2 or ≥3). Others included the PrEP introduction method (clinic-based or community-based), disclosure of same sexual practices to family (yes or no), disclosure of same sexual practices to health care workers (yes or no), and study time–months since PrEP initiation (3 or 9).

### Statistical analysis

**Fig 1:**
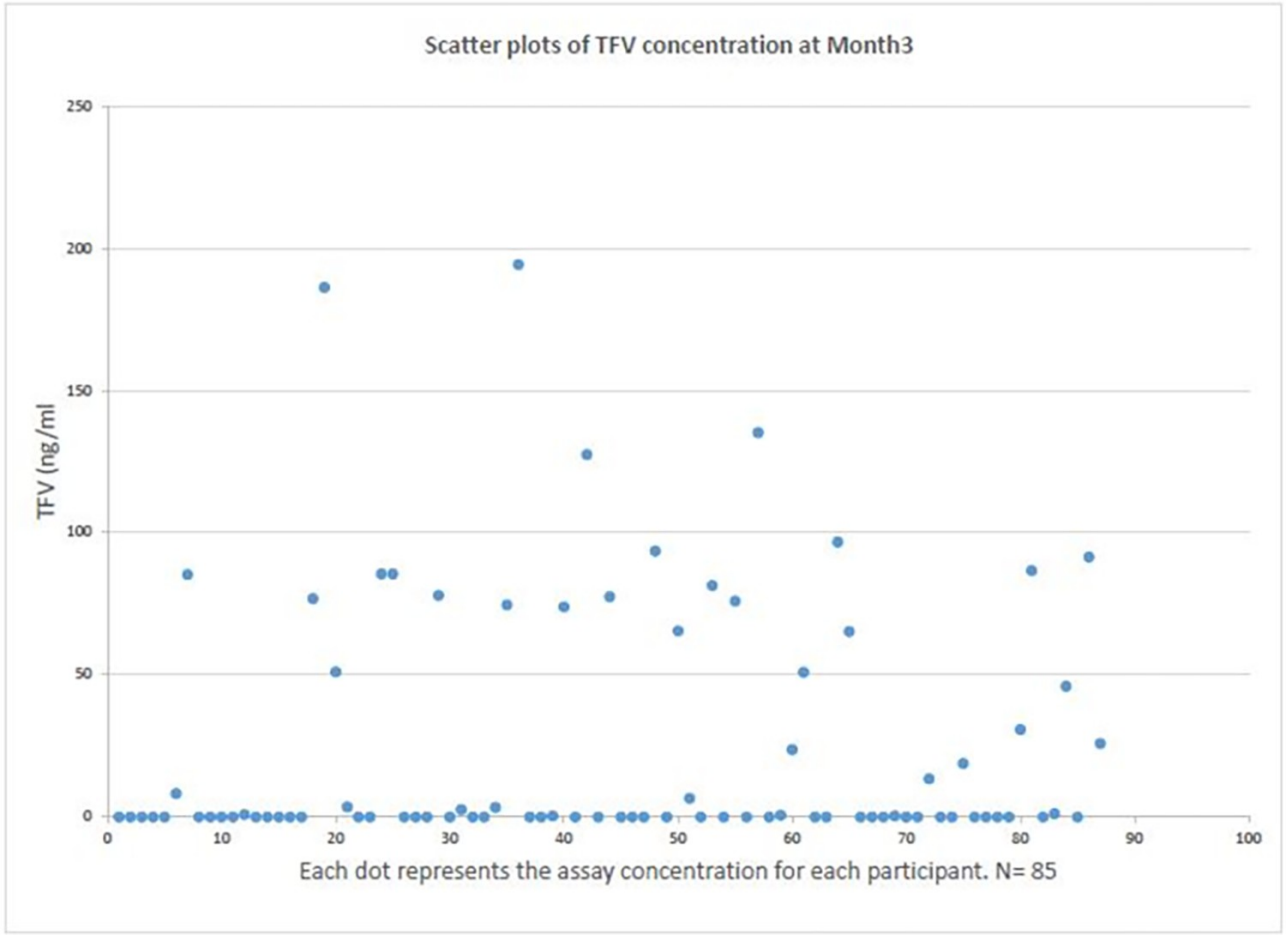
Scatter plots of absolute TFV concentration at Month 3.

**Fig 2:**
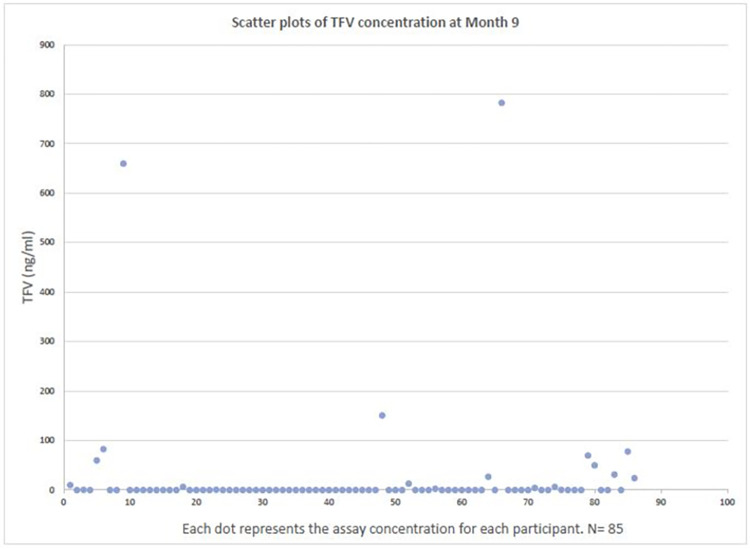
Scatter plots of absolute TFV concentration at Month 9.

We conducted univariable analyses for each variable. We plotted scatter plots of TFV concentration in absolute values at month 3 and 9 before we decided to use a cube-rooted value. (See Figs 1 and 2) To categorize self–reported PrEP adherence as a binary variable, an area under the curve (AUC) was estimated using a receiver operating characteristic (ROC) curve to evaluate how accurately each level of self–reported adherence predicted protective TFV concentration. The Youden’s index (which measures the maximum vertical distance between the ROC curve and the diagonal line) was used to determine the self-report level with the optimal cut-off point for protective TFV concentration [47]. Hence, "*very good*", and "*good*" were categorized as "Better than poor" and "*poor*" as Poor". We compared demographic, behavioral, and clinical characteristics between self-reported “Better than Poor” and self-reported “Poor” respondents using Pearson’s chi-square and fisher’s exact test for categorical variables with a bivariate analysis. We sought to determine each variable’s association with the self-reported PrEP adherence using logistic regression to estimate crude odds ratios and 95% confidence intervals. A partial Spearman’s rank correlation test, controlling for significant demographic variables, was utilized to examine the correlation between the biomarker PrEP assay and the self-reported PrEP adherence at month 3 and 9 visits, respectively. A p-value of < 0.05 was considered significant.

To assess for potential effect modifiers, a potential interaction variable (age) was selected *a priori*, and an interaction term was created with the self-reported PrEP adherence (primary exposure variable). A p-value of < 0.05 was considered significant. A variable needed to meet at least three of the following five criteria to be included in the multivariable analysis as a confounder. An association with the exposure variable (p ≤ 0.05), the outcome (p ≤ 0.20), a known confounder in the literature, identification by directed acyclic graph (DAG) as a minimal set of priori confounders, or a change in the crude regression coefficient by at least 10%. We added variables to the model one at a time and retained if significant. The study time, "months since PrEP initiation" variable was added based on prior knowledge of the variable’s importance.

After testing the model fits of different within and between subject correlation specifications, a generalized estimating equation for binary data with Logit link function (GEE) with an independent correlation structure was the most parsimonious correlation structure option. However, GEE sandwich variance estimates are robust and can correct most correlation mis-specifications [48]. A complete case analysis was conducted for the Spearman’s correlation, all variables missing outcomes were treated with list-wise deletion and “last observed carried forward” for those missing exposure variables [49]. For other missing variables, sensitivity analysis was conducted. Statistical analyses were conducted using SAS version 9.4 (SAS Institute Inc, Cary, NC, USA).

## Results

**Fig 3:**
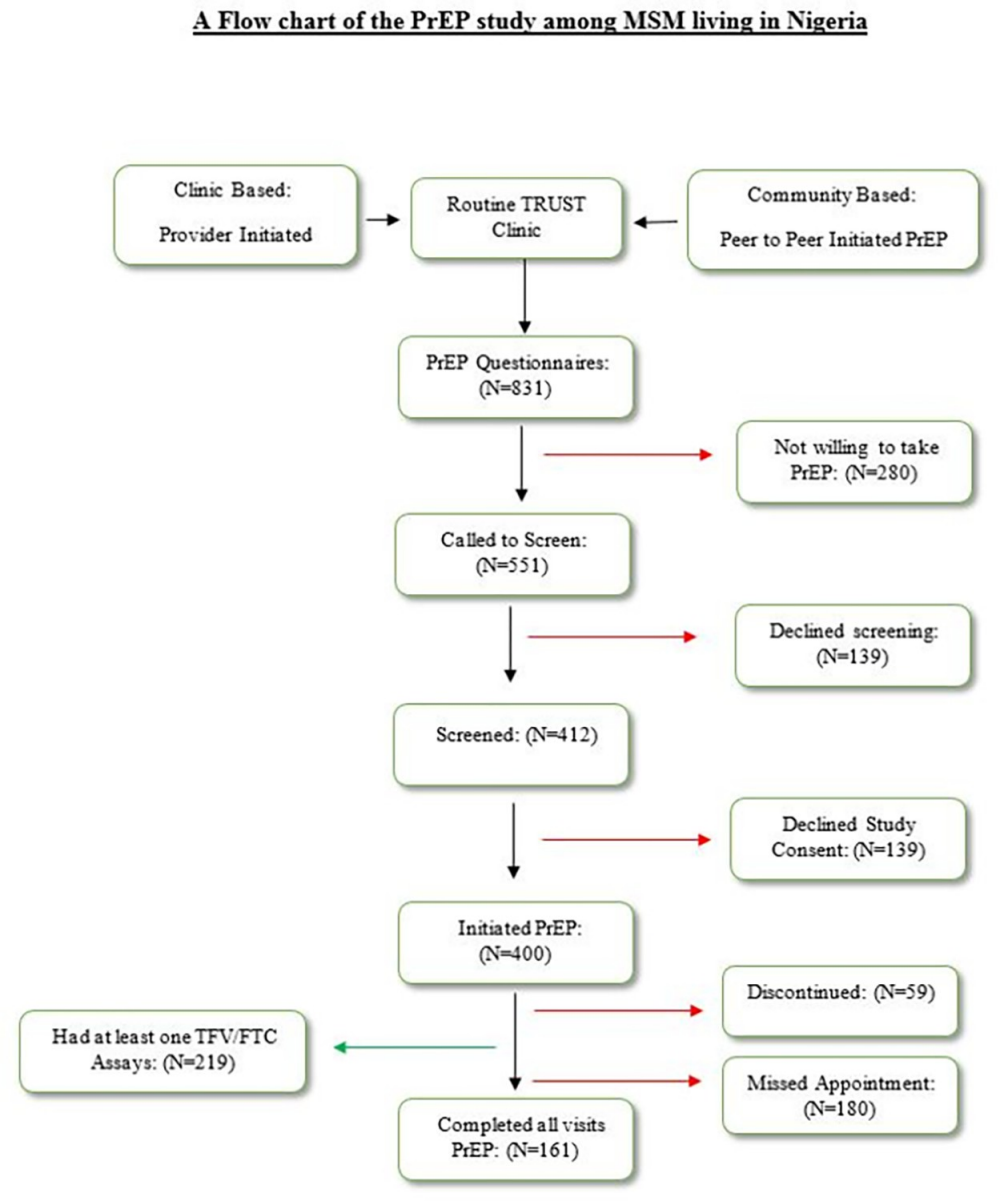
Study flowchart.

A total of 219 MSM participants between the ages of 17 and 54 years had at least one sample available for bioanalysis (See Fig 3 for study flowchart). Their median age was 23 years [interquartile range (IQR) 20–27 years]. A slight majority of participants (54.6%) were introduced to PrEP via the clinic-based approach. Nearly two-thirds (60.6%) had at least a high school education, 82% were employed, 85.5% self-identified as male, while 79.4% were bi-sexual. Almost all (98.9%) were never married.

The mean AUC for the ROC for the model used to evaluate how accurately each level of self–reported adherence predicted protective TFV concentration was 0.60. The mean sensitivity for prediction of protective concentration for respondent who reported poor, good and very good were 0.02, 0.84 and 0.95 respectively. The Youden’s index was -0.05, 0.10 and 0 respectively.

**Fig 4:**
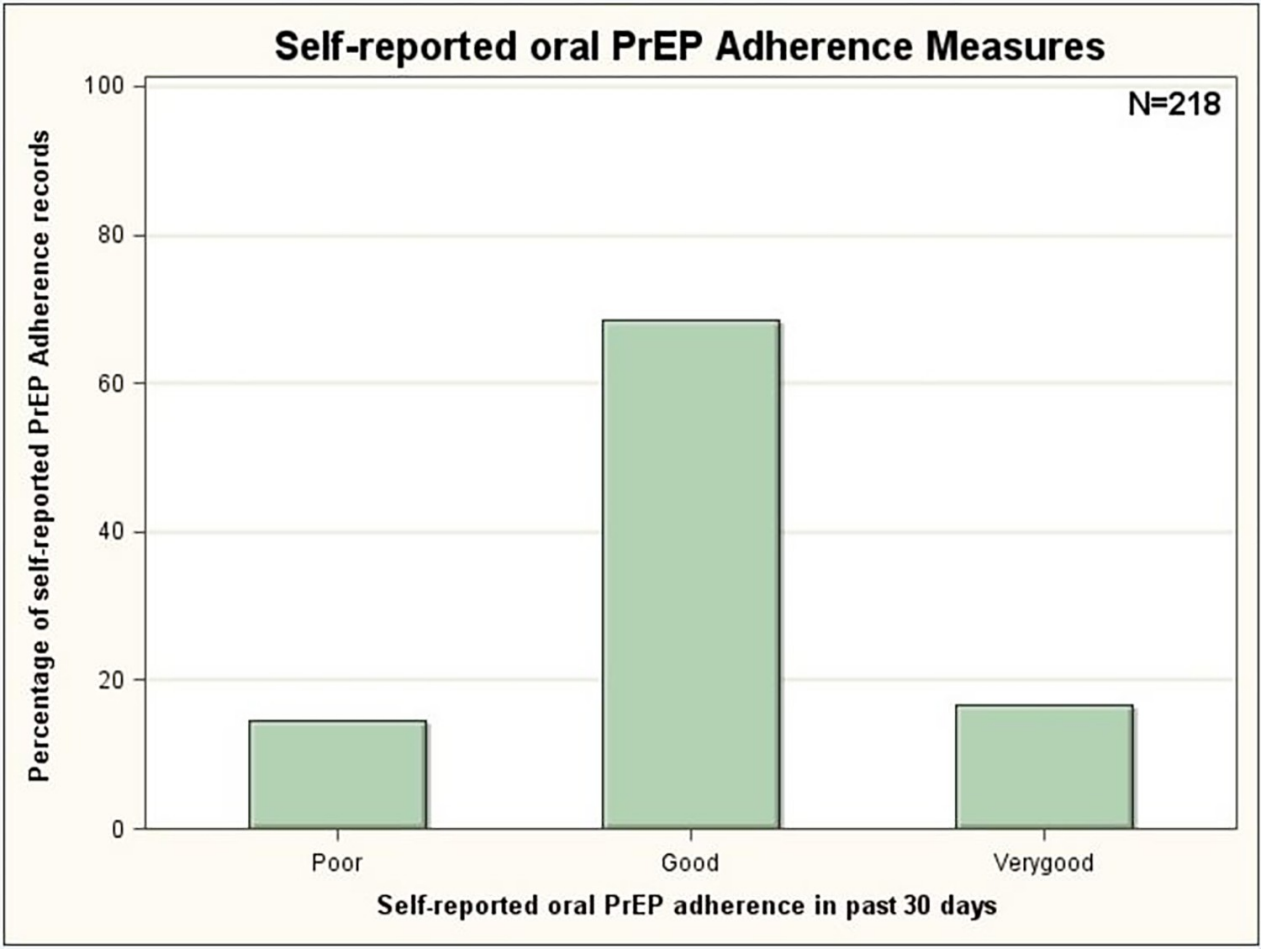
Summary of self-reported PrEP adherence measures in past 30 days.

**Fig 5:**
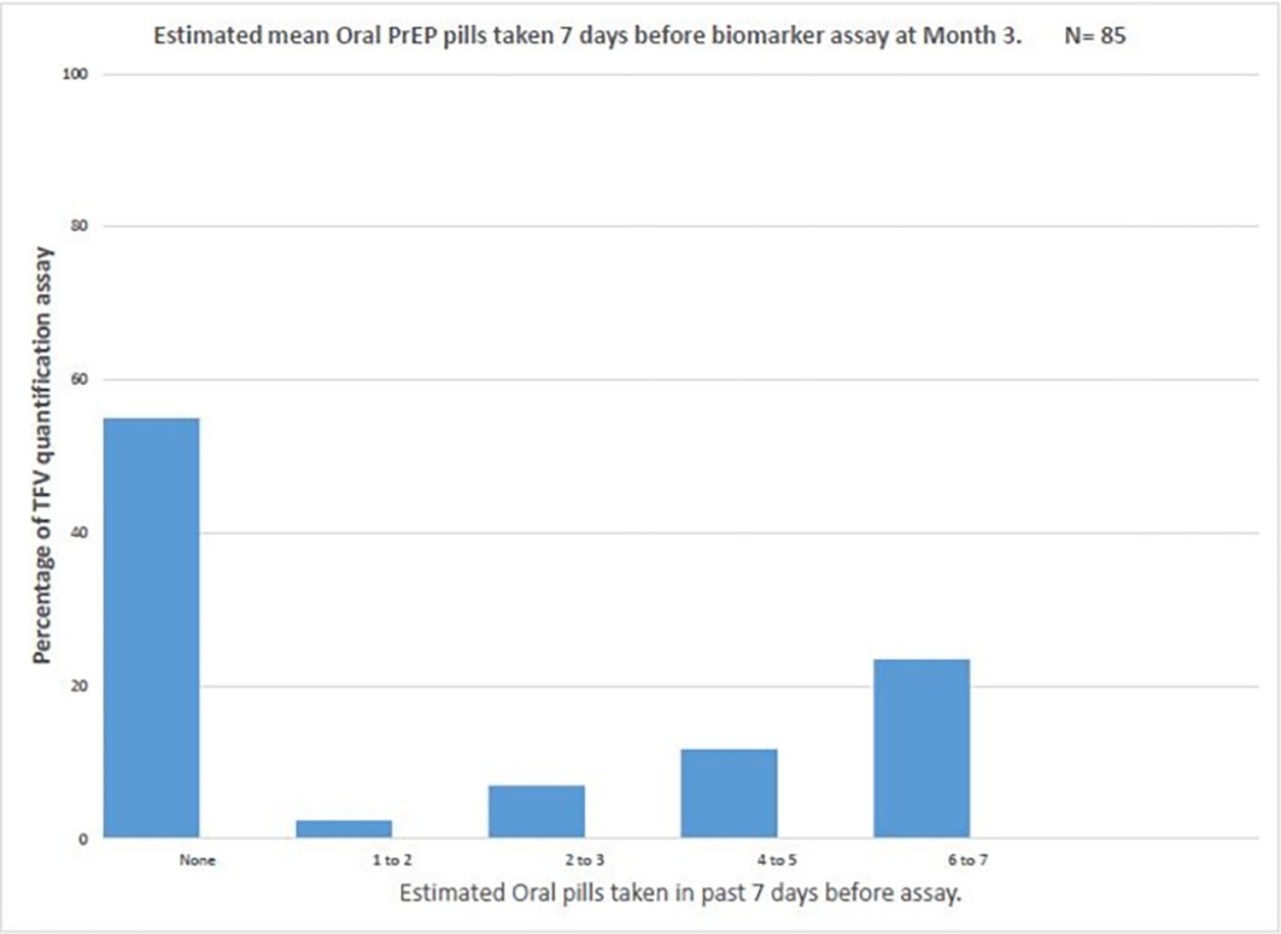
Estimated mean oral PrEP pills used a week before assay at Month 3.

**Fig 6:**
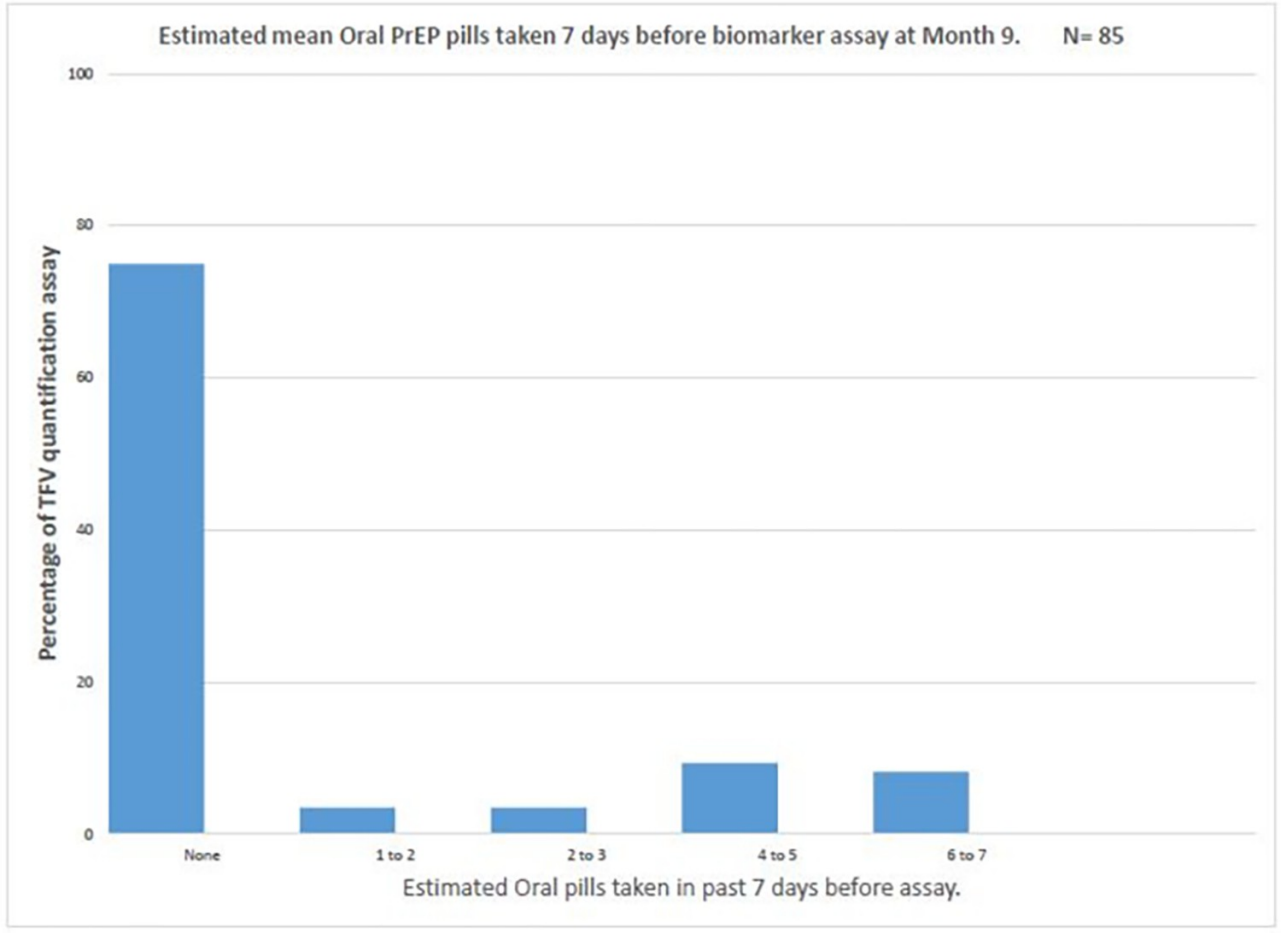
Estimated mean oral PrEP pills used a week before assay at Month 9.

Over the course of the study, 81 (37%) had a quantifiable assay (TFV ≥ 0.31 ng/mL); however, only [66/219] 30% and [49/219] 22% had TFV concentrations > 4.2 ng/mL and > 35.5 ng/ml, suggesting protective TFV concentration and daily adherence, respectively. Overall, there were 93 paired and 128 unpaired assays. An assay was paired if a respondent had at least two specimens at different study visits. The majority ([85/93] 91.3%) of the paired assays were between months 3 and 9. Comparing all paired assays, there was a significant negative change (Z = -2.60, p = 0.01) between those with protective TFV concentration at month 3, [30/85] 35% and those with protective TFV concentration at month 9, [15/85] 18%. All paired assays with no changes (N = 42/85) had below limit of assay quantification in both visits. Of those participants with self-reported PrEP adherence records, [176/218] 81% reported at least "good" PrEP adherence over the past month of their interview date (Fig 4). Of all PrEP adherence self-reports uniquely paired with identified biomarkers assays on any study visits [120/176] 68% objectively over-reported their PrEP adherence. In the immediate past week before assay, most participants (>60%) did not take any pill (Figs 5 and 6).

In bivariable analyses stratified by last reported self-reported PrEP adherence status, there was no difference in characteristics (Table 1).

**Table 1:** Characteristics of respondents stratified by last reported self-reported adherence status.

Characteristics	Total	SRPA (Better than poor)	SRPA (Poor)	p-value
	N = 218	N = 176	N = 42	
	n (%)	n (%)	n (%)	
**Age (Years)**				
Older (≥25)	100 (45.9)	77 (43.7)	23 (54.8)	0.20
Younger (16–24)	118 (54.1)	99 (56.3)	19 (45.2)	
**# Education**				
≤ High school	80 (60.6)	68 (64.2)	12 (46.2)	0.09
> High School	52 (39.4)	38 (35.8)	14 (53.8)	
**Employment**				
Unemployed	39 (17.9)	30 (17.0)	9 (21.4)	0.50
Employed	179 (82.1)	146 (83.0)	33 (78.6)	
**# Sexual orientation**				
Homosexual	27 (20.6)	20 (18.9)	7 (28.0)	0.30
Bisexual	104 (79.4)	86 (81.1)	18 (72.0)	
**# Gender identity**				
Cisgender man	112 (85.5)	92 (86.8)	20 (80.0)	0.36†
Transgender woman	19 (14.5)	14 (13.2)	5 (20.0)	
**# Condom use(last anal sex)**				
Yes	124 (68.9)	101 (69.2)	23 (67.6)	0.86
No	56 (31.1)	45 (30.8)	11 (32.4)	
**# Concurrency (males only)**				
Yes	66 (36.7)	56 (38.4)	10 (29.4)	0.32
No	114 (63.3)	90 (61.6)	24 (70.6)	
**# Alcohol in the last 30 days**				
0–2 drinks	74 (56.0)	60 (56.1)	14 (56.0)	0.99
≥3 drinks	58 (44.0)	47 (43.9)	11 (44.0)	
**Disclosure of Same-Sex Sexual Practices to Family**				
Yes	16 (7.3)	14 (8.0)	2 (4.8)	0.47†
No	202 (92.7)	162 (92.0)	40 (95.2)	
**Disclosure of Same-Sex Sexual Practices to Healthcare Workers**				
Yes	75 (34.4)	60 (34.1)	15 (35.7)	0.84
No	143 (65.6)	116 (65.9)	27 (64.3)	
**Months since PrEP initiation**				
3	64 (29.4)	49 (27.8)	15 (35.7)	0.31
9	154 (70.6)	127 (72.2)	27 (64.3)	
**PrEP Introduction Method**				
Community-based	99 (45.4)	81 (46.0)	18 (42.9)	0.71
Clinic-based	119 (54.6)	95 (54.0)	24 (57.1)	

Pearson chi-square and Fisher’s exact test [†] were used for categorical variables and categorical variables with expected cell count less than 5 respectively.

“[#]” indicates that the number of participants analyzed does not add up to N = 218.

Missing Variables: Education (86), Sexual Orientation (87), Gender identity (87), Condom use (38), Concurrency (38), Alcohol in the last 30 days (86).

After controlling for age, education, and employment, the adjusted spearman’s rank correlation between ordered category (poor, good and very good) self-reported PrEP adherence and TFV quantification assay (ng/mL, cube-rooted) at months 3 and 9 were 0.1 (95% CI: -0.12, 0.31) and 0.02 (95% CI: -0.20, 0.20), respectively. Table 2 shows more details of the correlations.

**Table 2:** Spearman correlation coefficient of quantification of TFV-FTC and self-reported adherence measures.

Spearman correlation coefficients of quantification of TFV-FTC [ng/ml, cube-rooted] and self-reported adherence measures [ranked poor, good and very good].				
	Serum TFV (month 3)	Serum FTC (month 3)	Serum TFV (month 9)	Serum FTC (month 9)
	ῤ (95% CI)	ῤ (95% CI)	ῤ (95% CI)	ῤ (95% CI)
	N [p-value]	N [p-value]	N [p-value]	N [p-value]
	GM: 2.93	GM: 3.37	GM: 2.62	GM: 2.27
Self- Reported Adherence (month 3) N	0.1 (-0.12,0.31)	0.2 (-0.04, 0.38)	0.1 (-0.08, 0.34)	0.2 (-0.01,0.40)
	81 [0.38]	81 [0.10]	81 [0.23]	81 [0.06]
Self- Reported Adherence (month 9) N	0.04 (-0.17, 0.24)	0.08 (-0.13, 0.29)	0.02(-0.20,0.2)	0.08(-0.14, 0.28)
	81 [0.74]	85 [0.46]	85 [0.89]	85 [0.49]

N: Number of observations. 95% CIs were calculated using Fisher’s transformation method.

ῤ: Correlation coefficients. GM: Geometric Mean (in nanogram per milliliters, cube-rooted)

Some covariates were independently associated with protective TFV concentration in this study (Table 3). Compared with participants receiving community-based PrEP introduction, those with clinic-based PrEP introduction were more likely to have achieved protective TFV concentration (OR: 5.40, 95% CI [2.67, 10.92]). Compared with those who did not disclose same sex practices to family members, those that disclosed were more likely to have achieved protective TFV concentration (OR: 2.72, 95% CI [1.29, 5.72]).

**Table 3:** GEE analysis of self-reported adherence and protective TFV concentration.

Characteristics	Crude OR (95% CI)	p- value	Adjusted OR (95% CI)	p- value
**Self-reported PrEP adherence**				
Poor	Ref		Ref	
Better than Poor	1.96 (0.84, 4.53)	0.12	2.10 (0.85, 5.18)	0.11
**PrEP Introduction Method**				
Community-based	Ref		**Ref**	
Clinic-based	**5.40 (2.67, 10.92)**	**<0.01**	**8.35 (3.24, 21.52)**	**<0.01**
**Age (Years)**				
Older (≥25)	Ref		Ref	
Younger (16–24)	0.58 (0.33, 1.03)	0.06	1.02 (0.51, 2.05)	0.95
**Condom Use (last anal sex)**				
Yes	**Ref**		Ref	
No	**1.83 (1.20, 2.79)**	< 0.01	1.65 (0.99, 2.74)	0.05
**Disclosure of Same-Sex Sexual Practices to Family**				
No	Ref		**Ref**	
Yes	**2.72 (1.29, 5.72)**	**0.01**	**3.60 (1.73, 7.51)**	**<0.01**
**Months since PrEP initiation**				
3	Ref		Ref	
9	1.61 (0.80, 3.20)	0.18	2.30 (0.98, 5.38)	0.06

Generalized Estimating Equations with an independent correlation structure was used for multivariable analysis. The model was adjusted for the PrEP introduction method, participants’ age, disclosure of sexual orientation to family members, condom use during last sex with a male partner, and time of the study.

**Bolded** confidence intervals indicate significance at p < 0.05.

Definitions

Protective TFV Concentration: Serum TFV concentration ≥ 4.2 ng/ml

Self-reported PrEP Adherence: Poor (Those that reported poor) | Better than Poor(Those that reported good, very good or perfect)

In multivariable GEE analysis (Table 3), after adjusting intra and between subject correlations, PrEP introduction method and disclosure of sexual orientation to family members remained significantly associated with protective TFV concentration.

Regarding missingness, an average of 9% of all variables were missing in our study. Participants with missing “alcohol in the last 30 days” variables were more likely to have had protective TFV concentration PrEP adherence than those not missing those variables in this study.

## Discussion

There was no significant correlation between self-reported PrEP adherence and quantitative measures of PrEP biomarkers in our study. However, other findings provided insight into PrEP adherence measures within the context of resource-limited settings such as in Nigeria. Of note, in this study, more than two-thirds of study participants over-reported their PrEP adherence. The two studies that investigated self-reported PrEP adherence we found from Africa were based in Kenya and Uganda [37, 50]. Similar to our study, the participants (although not exclusively MSM) over-reported their PrEP adherence [50]. In other studies from high income settings, [25, 51], a median of 40% and 75% MSM respectively over-reported their PrEP adherence in the Adolescent Medicine Trial Network (ATN110 and 113) daily oral PrEP safety and efficacy trials in 16 urban sites in the United States.

We found that participants who had clinic-based PrEP introduction were more likely to have achieved protective TFV concentration. A possible explanation for better adherence among participants with the clinic-based approach may be that the TRUST clinic provided a safer, more discreet space and more conducive environment for PrEP introduction counselling, as compared to counselling undertaken within the community. Prior data have shown that engagement in care at trusted community health centers in Nigeria devoid of same sexual relationship stigmatization such as the TRUST clinic in our study, had beneficial effects that includes, but not limited to increased HIV-related knowledge [52] and improved uptake of condoms and condom-compatible lubricants [53]. Clinical providers in these unique settings have baseline and continuous routine refresher courses on patient centered approaches, hence, this might explain the better outcomes as compared with the POLs who introduced PrEP by the community-based method. Differentiated service delivery that considers and respects the unique needs of sexual and gender minority populations may improve PrEP adherence and other health outcomes.

Another factor associated with increased likelihood of protective TFV concentration in our study was disclosure of same-sex sexual practices to family members. In our study, fewer than 10% of all participants disclosed same sex practices to their family. Such disclosures are challenging in rights-constrained settings like Nigeria. However, prior data have shown that this type of disclosure is associated with improved health behaviors such as increased condom usage [54] and better adherence to ART [55]. Similarly, among people living with HIV, disclosure of HIV status to sexual partners is associated with an increased likelihood of viral suppression [56]. Together, these data suggest that making family members and sexual partners part of the discussion surrounding safer sex practices may facilitate adherence.

Participants’ age was not significantly associated with protective TFV concentration in our study. This is similar to findings in other studies from Kenya, Uganda, Burkina Faso, Cote d’Ivoire, Mali and Togo where age was not reported to have been associated with protective TFV concentration [21, 22, 37, 57]. However, our findings are unlike those from the US PrEP demonstration project [58], the HIV Prevention Trials Network (HPTN) 067 studies [33], and the ATN 100 and ATN 113 trials [25] where older age had been associated with better PrEP adherence among MSM. This finding in our study might be because there was not enough variation in our study participants’ ages as more than 75% were under 30 years. There might be a need to conduct more studies in similar settings to have a better insight of the impact of age on PrEP adherence.

In contrast to our hypothesis, we found no significant discordance in self-reported PrEP adherence between the month 3 and month 9 study visits. In previous studies [40, 51], the average correlation between these two adherence methods was between 0.25 and 0.4, but this was much lower (0.02 to 0.2) in our study. The lower correlation in our study may be explained by a relatively higher proportion of non-adherence (Almost two-thirds [63%] of participants had unquantifiable TFV concentrations) as compared to previous studies. A similar trend of low TFV assay among MSM taking PrEP was reported in two studies [21, 22] from Kenya where only 14.7% and 14.5% had any TFV detection and protective TFV concentration respectively. This inconsistency even within the same individuals longitudinally underscores the unreliability of self-reported adherence data in our study. A mixed-method study identified potential PrEP information enablers among key population in Nigeria [20]. One of them was the use of reminders. Efforts should be geared towards youth friendly platform reminders to improve adherence [59].

Previous studies [31, 34, 46, 60] have demonstrated that serum and or plasma TFV measurements are useful for investigating recent (within the past 7 days) PrEP adherence. We, therefore, sought to leverage this to investigate our study participants’ immediate past week adherence (Figs 5 and 6). It could be inferred from the TFV serum assay concentrations that the majority of the participants (63%) did not take any TDF pill within the immediate past week, while ~7% took a sub-optimal (1–3 pills) dose. Interestingly, among the 30% with protective TFV concentration, almost two-thirds had a daily adherence within the last week of assay quantification. Although this might be a consequence of white coat adherence [27, 32, 61]. The use of DBS would have provided a longer window of PrEP detectability, as the half-life of tenofovir diphosphate (TFV-DP) in erythrocytes is much longer [28] than TFV in systemic compartments; it should be noted that dried blood spots (DBS) were not collected in this study.

This study had several limitations. We had an average of 9% missingness in our study variables. However, we performed a sensitivity analysis to determine the best way to handle and interpret our study findings and found that all variables except alcohol use were missing completely at random, relative to the observed (outcome) variables. Therefore, our study variables’ missingness is not expected to impact the interpretation of our analysis. Another limitation was the low event-to-trial outcome that may have underpowered our study. We could not rule out potential selection bias that might have resulted from attrition of participants in our study. (See Fig 3).

Notwithstanding these limitations, this study used an objective biomedical measure to determine PrEP use. Furthermore, this study has added to our understanding of implementing PrEP among a population residing in rights-constrained settings.

## Conclusion

In this study, we found that self-reported PrEP adherence measure was a poor measure of PrEP adherence. In addition, the settings for PrEP introduction and the disclosure of same-sex sexual practices to family members seemed to have impacted PrEP adherence among MSM in Nigeria. While other HIV program areas might have successfully engaged peer-to-peer counselling, care ought be taken to carefully study and contextualize these into the uniqueness of a vulnerable, mobile, and hard-to-reach population living in a MSM-hostile environment. Programs implementing PrEP in similar settings might want to conduct further studies on factors associated with PrEP adherence. In future studies, we recommend a post-PrEP adherence qualitative study comparing shared experiences of the adherent and non-adherent participants. Finally, the recent FDA approval of long acting injectable PrEP [62] may make way for a more discreet option that might overcome the barriers associated with oral PrEP. This may alter the landscape of PrEP adherence in the African settings as reported in South Africa [36, 63].

## Supporting information

S1 Checklist Inclusivity in global research. (DOCX)
